# Bioactive Compounds and Bioactivities of Ginger (*Zingiber officinale* Roscoe)

**DOI:** 10.3390/foods8060185

**Published:** 2019-05-30

**Authors:** Qian-Qian Mao, Xiao-Yu Xu, Shi-Yu Cao, Ren-You Gan, Harold Corke, Trust Beta, Hua-Bin Li

**Affiliations:** 1 Guangdong Provincial Key Laboratory of Food, Nutrition and Health, Department of Nutrition, School of Public Health, Sun Yat-sen University, Guangzhou 510080, China; maoqq@mail2.sysu.edu.cn (Q.-Q.M.); xuxy53@mail2.sysu.edu.cn (X.-Y.X.); caoshy3@mail2.sysu.edu.cn (S.-Y.C.); 2 Department of Food Science & Technology, School of Agriculture and Biology, Shanghai Jiao Tong University, Shanghai 200240, China; hcorke@sjtu.edu.cn; 3 Department of Food & Human Nutritional Sciences, University of Manitoba, Winnipeg, MB R3T 2N2, Canada; Trust.Beta@umanitoba.ca; 4 Richardson Centre for Functional Foods and Nutraceuticals, University of Manitoba, Winnipeg, MB R3T 2N2, Canada

**Keywords:** phytochemicals, antioxidant, antinausea, antiobesity, anticancer, anti-inflammatory

## Abstract

Ginger (*Zingiber officinale* Roscoe) is a common and widely used spice. It is rich in various chemical constituents, including phenolic compounds, terpenes, polysaccharides, lipids, organic acids, and raw fibers. The health benefits of ginger are mainly attributed to its phenolic compounds, such as gingerols and shogaols. Accumulated investigations have demonstrated that ginger possesses multiple biological activities, including antioxidant, anti-inflammatory, antimicrobial, anticancer, neuroprotective, cardiovascular protective, respiratory protective, antiobesity, antidiabetic, antinausea, and antiemetic activities. In this review, we summarize current knowledge about the bioactive compounds and bioactivities of ginger, and the mechanisms of action are also discussed. We hope that this updated review paper will attract more attention to ginger and its further applications, including its potential to be developed into functional foods or nutraceuticals for the prevention and management of chronic diseases.

## 1. Introduction

Ginger (*Zingiber officinale* Roscoe), which belongs to the Zingiberaceae family and the *Zingiber* genus, has been commonly consumed as a spice and an herbal medicine for a long time [1]. Ginger root is used to attenuate and treat several common diseases, such as headaches, colds, nausea, and emesis. Many bioactive compounds in ginger have been identified, such as phenolic and terpene compounds. The phenolic compounds are mainly gingerols, shogaols, and paradols, which account for the various bioactivities of ginger [2]. In recent years, ginger has been found to possess biological activities, such as antioxidant [3], anti-inflammatory [4], antimicrobial [5], and anticancer [6] activities. In addition, accumulating studies have demonstrated that ginger possesses the potential to prevent and manage several diseases, such as neurodegenerative diseases [7], cardiovascular diseases [8], obesity [9], diabetes mellitus [10], chemotherapy-induced nausea and emesis [11], and respiratory disorders [12]. In this review, we focus on the bioactive compounds and bioactivities of ginger, and we pay special attention to its mechanisms of action.

## 2. Bioactive Components and Bioactivities of Ginger

### 2.1. Bioactive Components

Ginger is abundant in active constituents, such as phenolic and terpene compounds [13]. The phenolic compounds in ginger are mainly gingerols, shogaols, and paradols. In fresh ginger, gingerols are the major polyphenols, such as 6-gingerol, 8-gingerol, and 10-gingerol. With heat treatment or long-time storage, gingerols can be transformed into corresponding shogaols. After hydrogenation, shogaols can be transformed into paradols [2]. There are also many other phenolic compounds in ginger, such as quercetin, zingerone, gingerenone-A, and 6-dehydrogingerdione [14,15]. Moreover, there are several terpene components in ginger, such as β-bisabolene, α-curcumene, zingiberene, α-farnesene, and β-sesquiphellandrene, which are considered to be the main constituents of ginger essential oils [16]. Besides these, polysaccharides, lipids, organic acids, and raw fibers are also present in ginger [13,16].

### 2.2. Antioxidant Activity

It has been known that overproduction of free radicals, such as reactive oxygen species (ROS), plays an important part in the development of many chronic diseases [17]. It has been reported that a variety of natural products possess antioxidant potential, such as vegetables, fruits, edible flowers, cereal grains, medicinal plants, and herbal infusions [18,19,20,21,22,23,24]. Several studies have found that ginger also has high antioxidant activity [14,25]. 

The antioxidant activity of ginger has been evaluated in vitro via ferric-reducing antioxidant power (FRAP), 2,2-diphenyl-1-picrylhydrazyl (DPPH), and 2,2′-azinobis-(3-ethylbenzothiazoline-6-sulfonic acid) (ABTS) methods. The results revealed that dried ginger exhibited the strongest antioxidant activity, because the number of phenolic compounds was 5.2-, 1.1-, and 2.4-fold higher than that of fresh, stir-fried, and carbonized ginger, respectively. The antioxidant activity of different gingers had a tendency to be the following: dried ginger > stir-fried ginger > carbonized ginger > fresh ginger. This was mainly associated with their polyphenolic contents. When fresh ginger was heated, dried ginger with higher antioxidant activity was obtained, because fresh ginger contains a higher moisture content. However, when dried ginger was further heated to obtain stir-fried ginger and carbonized ginger, the antioxidant activity decreased, because the processing could change gingerols into shogaols [26]. Additionally, a fraction of the dried ginger powder abundant in polyphenols showed high antioxidant activity based on data from FRAP, oxygen radical absorbance capacity, and cellular antioxidant activity assays [27]. Besides, the type of extraction solvent could have an effect on the antioxidant activity of ginger. An ethanolic extract of ginger showed high Trolox-equivalent antioxidant capacity and ferric-reducing ability, and an aqueous extract of ginger exhibited strong free radical scavenging activity and chelating ability [16]. Moreover, ethanolic, methanolic, ethyl acetate, hexane, and water extracts of ginger respectively inhibited 71%, 76%, 67%, 67%, and 43% of human low-density lipoprotein (LDL) oxidation induced by Cu^2+^ [28]. Results from a xanthine/xanthine oxidase system showed that an ethyl acetate extract and an aqueous extract had higher antioxidant properties than ethanol, diethyl ether, and *n*-butanol extracts did [3].

**Figure 1:**
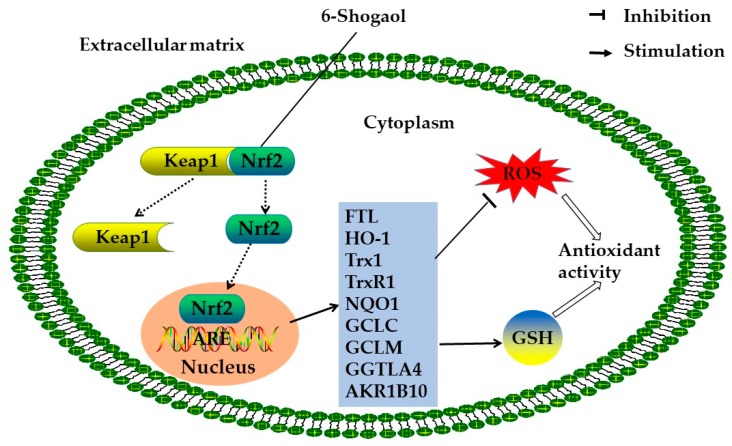
The potential mechanism for the antioxidant action of 6-shogoal: 6-shogoal leads to the translocation of Nrf2 into the nucleus and increases the expression of Nrf2 target genes by modifying Keap1 and preventing Nrf2 from proteasomal degradation. Thus, the level of GSH increases, and the level of ROS decreases. Abbreviations: Nrf2, nuclear factor erythroid 2-related factor 2; Keap1, Kelch-like ECH-associated protein 1; *NQO1*, nicotinamide adenine dinucleotide phosphate (NADPH) quinone dehydrogenase 1; *HO-1*, heme oxygenase-1; *GCLC*, glutamate-cysteine ligase catalytic subunit; *GCLM*, glutamate-cysteine ligase modifier subunit; *Trx1*, thioredoxin 1; *TrxR1*, thioredoxin reductase 1; *AKR1B10,* Aldo-keto reductase family 1 member B10; *FTL*, ferritin light chain; *GGTLA4*, γ-glutamyltransferase-like activity 4; ROS, reactive oxygen species; GSH, glutathione; ARE, antioxidant response element.

Several studies have indicated that ginger was effective for protection against oxidative stress. The underlying mechanisms of antioxidant action were investigated in cell models [14,29]. Ginger extract showed antioxidant effects in human chondrocyte cells, with oxidative stress mediated by interleukin-1β (IL-1β). It stimulated the expression of several antioxidant enzymes and reduced the generation of ROS and lipid peroxidation [30]. Additionally, ginger extract could reduce the production of ROS in human fibrosarcoma cells with H_2_O_2_-induced oxidative stress [31]. In stressed rat heart homogenates, ginger extract decreased the content of malondialdehyde (MDA), which was related to lipid peroxidation [29]. Ginger and its bioactive compounds (such as 6-shogaol) exhibited antioxidant activity via the nuclear factor erythroid 2-related factor 2 (Nrf2) signaling pathway (Figure 1) [32]. In human colon cancer cells, 6-shogaol increased intracellular glutathione/glutathione disulfide (GSH/GSSG) and upregulated Nrf2 target gene expression, such as with heme oxygenase-1 (*HO-1)*, metallothionein 1 (*MT1*), aldo-keto reductase family 1 member B10 (*AKR1B10*), ferritin light chain (*FTL*), and γ-glutamyltransferase-like activity 4 (*GGTLA4*). Besides, 6-shogaol also enhanced the expression of genes involved in glutathione synthesis, such as the glutamate-cysteine ligase catalytic subunit (*GCLC*) and the glutamate-cysteine ligase modifier subunit (*GCLM*). Further analysis revealed that 6-shogaol and its metabolite activated Nrf2 via the alkylation of cysteine residues of Kelch-like ECH-associated protein 1 (Keap1) [33]. Moreover, ginger phenylpropanoids improved Nrf2 activity and enhanced the levels of glutathione S-transferase P1 (GSTP1) as well as the downstream effector of the Nrf2 antioxidant response element in foreskin fibroblast cells [15]. In a human mesenchymal stem cell model, ginger oleoresin was investigated for its effects on injuries that were induced by ionizing radiation. The treatment of oleoresin could decrease the level of ROS by translocating Nrf2 to the cell nucleus and activating the gene expression of *HO-1* and *NQO1* (nicotinamide adenine dinucleotide phosphate (NADPH) quinone dehydrogenase 1) [14].

An animal model has also been used to investigate the antioxidant properties of ginger and its bioactive compounds in vivo. There, 6-shogaol exhibited antioxidant potential by inducing the expression of Nrf2 target genes such as *MT1*, *HO-1*, and *GCLC* in the colon of wild-type mice, but not Nrf2^−/−^ mice [33]. In addition, rats with a gastric ulcer induced by diclofenac sodium were treated with the butanol extract of ginger. It could prevent an increase in the level of MDA and a decrease in catalase activity as well as the level of glutathione [34]. Moreover, the 6-gingerol-rich fraction from ginger could reduce the levels of H_2_O_2_ and MDA, enhance antioxidant enzyme activity, and increase glutathione in rats with oxidative damage induced by chlorpyrifos [25]. Furthermore, treatment with ginger extract elevated the contents of antioxidants and testosterone in serum and protected rat testes from injuries in chemotherapy with cyclophosphamide [35].

Overall, in vitro and in vivo studies have demonstrated that ginger and its bioactive compounds, such as 6-shogaol, 6-gingerol, and oleoresin, possess strong antioxidant activity (Table 1). Moreover, the activation of the Nrf2 signaling pathway is crucial to the underlying mechanisms of action. It should also be pointed out that the overproduction of ROS in the human body is considered to be a cause of many diseases. Theoretically, antioxidants should be effective. However, several factors, such as health conditions, individual differences, the lifestyles of people, other dietary factors, and the dosage, solubility, and oral intake of antioxidants could affect the bioaccessibility and bioavailability of antioxidants, leading to low blood concentrations overall, which probably could explain why most antioxidants do not work in the real world.

### 2.3. Anti-Inflammatory Activity

A series of studies showed that ginger and its active constituents possessed anti-inflammatory activity (Table 2), which could protect against inflammation-related diseases such as colitis [4,36]. The anti-inflammatory effects were mainly related to phoshatidylinositol-3-kinase (PI3K), protein kinase B (Akt), and the nuclear factor kappa light chain-enhancer of activated B cells (NF-κB). 

In addition, 6-shogaol showed protective effects against tumor necrosis factor α (TNF-α)-induced intestinal barrier dysfunction in human intestinal cell models. It also prevented the upregulation of Claudin-2 and the disassembly of Claudin-1 via the suppression of signaling pathways involved with PI3K/Akt and NF-κB [37]. In addition, 6-dehydroshogaol was more potent than 6-shogaol and 6-gingerol in reducing the generation of proinflammatory mediators such as nitric oxide (NO) and prostaglandin E_2_ (PGE_2_) in mouse macrophage RAW 264.7 cells [36]. Besides, ginger extract and zingerone inhibited NF-κB activation and decreased the level of IL-1β in the colons of mice, which alleviated colitis induced by 2, 4, 6-trinitrobenzene sulfonic acid [38]. Ginger also protected against anti-CD3 antibody-induced enteritis in mice, and ginger could reduce the production of TNF-α as well as the activation of Akt and NF-κB [39]. Moreover, nanoparticles derived from edible ginger (GDNPs 2) could prevent intestinal inflammation by increasing the levels of anti-inflammatory cytokines such as interleukin-10 (IL-10) and IL-22 and decreasing the levels of proinflammatory cytokines such as TNF-α, IL-6, and IL-1β in mice with acute colitis and chronic colitis [4]. In addition, nanoparticles loaded with 6-shogaol were found to attenuate colitis symptoms and improve colitis wound repair in mice with dextran sulfate sodium-induced colitis [40]. Moreover, microRNAs of ginger exosome-like nanoparticles (GELN) ameliorated mouse colitis by inducing the production of IL-22, a barrier function improvement factor [41]. Additionally, a fraction rich in 6-gingerol prevented an increase in inflammatory markers such as myeloperoxidase, NO, and TNF-α in the brain, ovaries, and uterus of rats treated with chlorpyrifos [25]. Furthermore, 28 male endurance runners consumed capsules of 500 mg of ginger powder. The results showed that the treatment could attenuate the post-exercise elevation of several cytokines that promote inflammation, such as plasma IL-1β, IL-6, and TNF-α [42]. 

In general, ginger and its active compounds have been found to be effective in alleviating inflammation, especially in inflammatory bowel diseases. The anti-inflammatory mechanisms of ginger are probably associated with the inhibition of Akt and NF-κB activation, an enhancement in anti-inflammatory cytokines, and a decline in proinflammatory cytokines. Notably, the application of ginger nanoparticles has the potential to improve the prevention of and therapy for inflammatory bowel disease. 

### 2.4. Antimicrobial Activity

The spread of bacterial, fungal, and viral infectious diseases has been a major public threat due to antimicrobial resistance. Several herbs and spices have been developed into natural effective antimicrobial agents against many pathogenic microorganisms [43]. In recent years, ginger has been reported to show antibacterial, antifungal, and antiviral activities [44,45].

Biofilm formation is an important part of infection and antimicrobial resistance. One result found that ginger inhibited the growth of a multidrug-resistant strain of *Pseudomonas aeruginosa* by affecting membrane integrity and inhibiting biofilm formation [46]. In addition, treatment with ginger extract blocked biofilm formation via a reduction in the level of bis-(3′-5′)-cyclic dimeric guanosine monophosphate (c-di-GMP) in *Pseudomonas aeruginosa* PA14 [47]. Moreover, a crude extract and methanolic fraction of ginger inhibited biofilm formation, glucan synthesis, and the adherence of *Streptococcus mutans* by downregulating virulence genes. Consistent with the in vitro study, a reduction in caries development caused by *Streptococcus mutans* was found in a treated group of rats [48]. Furthermore, an in vitro study revealed that gingerenone-A and 6-shogaol exhibited an inhibitory effect on *Staphylococcus aureus* by inhibiting the activity of 6-hydroxymethyl-7, 8-dihydropterin pyrophosphokinase in the pathogen [49]. 

The compounds in ginger essential oil possess lipophilic properties, making the cell wall as well as the cytoplasmic membrane more permeable and inducing a loss of membrane integrity in fungi [50]. An in vitro study revealed that ginger essential oil effectively inhibited the growth of *Fusarium verticillioides* by reducing ergosterol biosynthesis and affecting membrane integrity. It could also decrease the production of fumonisin B_1_ and fumonisin B_2_ [51]. In addition, ginger essential oil had efficacy in suppressing the growth of *Aspergillus flavus* as well as aflatoxin and ergosterol production [50]. Moreover, the γ-terpinene and citral in ginger essential oil showed potent antifungal properties against *Aspergillus flavus* and reduced the expression of some genes related to aflatoxin biosynthesis [44]. Furthermore, fresh ginger was found to inhibit plaque formation induced by human respiratory syncytial virus (HRSV) in respiratory tract cell lines. Ginger was effective in blocking viral attachment and internalization [52]. In a clinical trial, ginger extract decreased hepatitis C virus (HCV) loads, the level of α-fetoprotein (AFP), and markers relevant to liver function, such as aspartate aminotransferase (AST) and alanine aminotransferase (ALT), in Egyptian HCV patients [53].

Therefore, ginger has been demonstrated to inhibit the growth of different bacteria, fungi, and viruses. These effects could be mainly related to the suppression of bacterial biofilm formation, ergosterol biosynthesis, and viral attachment and internalization (Table 3).

### 2.5. Cytotoxicity

**Figure 2:**
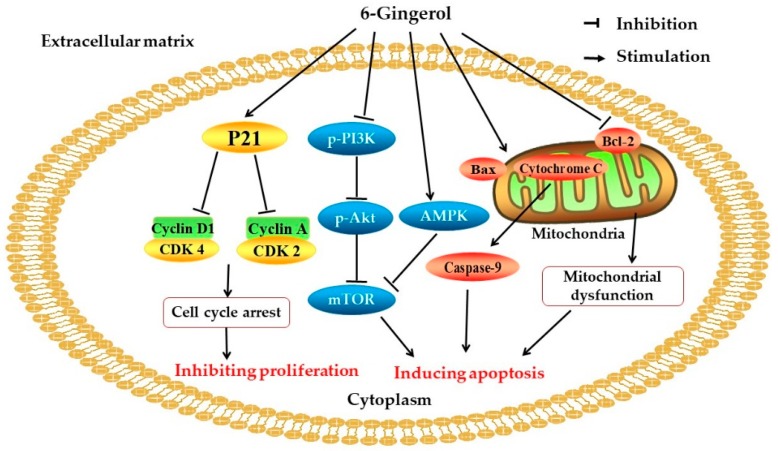
Several signaling pathways are involved in the anticancer mechanisms of 6-gingerol. CDK: Cyclin-dependent kinase; PI3K: Phosphoinositide 3-kinase; Akt: Protein kinase B; mTOR: Mammalian target of rapamycin; AMPK: 5’adenosine monophosphate-activated protein kinase; Bax: Bcl-2-associated X protein; Bcl-2: B-cell lymphoma 2.

Cancer is documented to be a dominant cause of death, and there were approximately 9.6 million cases of death in 2018 [54]. Several research works have demonstrated that natural products such as fruits and medicinal plants possess anticancer activity [55,56]. Recently, ginger has been widely investigated for its anticancer properties against different cancer types, such as breast, cervical, colorectal, and prostate cancer [4,57,58]. The potential mechanisms of action involve the inhibition of proliferation and the induction of apoptosis in cancer (Figure 2) [59,60]. 

Several investigations have demonstrated that ginger and its bioactive compounds can interfere with the carcinogenic processes of colorectal cancer. It was observed in an in vitro study that a fraction rich in the polyphenols of dried ginger powder suppressed the proliferation of colorectal cancer cells and gastric adenocarcinoma cells [27]. Besides, treatment with ginger extract promoted apoptosis by decreasing the expression of genes involved with the Ras/extracellular signal-regulated kinase (ERK) and PI3K/Akt pathways, such as the v-Ki-ras2 Kirsten rat sarcoma viral oncogene homolog (*KRAS*), *ERK, Akt*, and B-cell lymphoma-extralarge (*Bcl-xL*). It also increased the expression of caspase 9, which promoted apoptosis in HT-29 colorectal cancer cells [60]. In rats with 1,2-dimethylhydrazine-induced colon cancer, ginger extract loading with coated alginate beads increased the activities of NADH dehydrogenase and succinate dehydrogenase [61]. In addition, GDNPs 2 treatment decreased tumor numbers and tumor loads in mice with colitis-associated cancer induced by azoxymethane and dextran sodium sulfate. The levels of proinflammatory cytokines were decreased, and intestinal epithelial cell proliferation was inhibited [4]. In a pilot, randomized, and controlled trial, ginger extract supplementation decreased proliferation and increased apoptosis in the colonic mucosa of patients with a high risk of colorectal cancer. Ginger extract supplementation induced a decrease in the expression of two markers of cell proliferation, telomerase reverse transcriptase (hTERT) and MIB-1 (epitope of Ki-67), and increased the expression of pro-apoptotic gene Bcl-2-associated X (*Bax*) [6]. In subjects with a high risk of colorectal cancer, ginger supplementation decreased cyclooxygenase-1 (COX-1) expression, a key enzyme in the production of PGE_2_, which indicated the preventive potential of ginger in colorectal cancer [62].

The cytotoxic effects and underlying mechanisms of ginger in prostate cancer were evaluated both in vivo and in vitro. It was found that 6-gingerol, 10-gingerol, 6-shogaol, and 10-shogaol showed an antiproliferative effect on human prostate cancer cells via a downregulation of the protein expression of multidrug resistance associated protein 1 (MRP1) and glutathione-S-transferase (GSTπ) [59]. In addition, binary combinations of ginger phytochemicals, such as 6-gingerol, 8-gingerol, 10-gingerol, and 6-shogaol, synergistically inhibited the proliferation of PC-3 prostate cancer cells [63]. An in vivo study investigated the effect of ginger on athymic nude mice with human prostate tumor xenografts. A natural ginger extract showed a 2.4-fold higher inhibitory effect on the growth of tumors than an artificial mixture of 6-shogaol, 6-gingerol, 8-gingerol, and 10-gingerol [64]. Additionally, 6-shogaol could be more significant than 6-gingerol and 6-paradol in reducing cell survival and inducing apoptosis in human and mouse prostate cancer cells. It worked mainly through the suppression of signal transducer and activator of transcription 3 (STAT3) and NF-κB signaling. It also decreased the expression of *cyclinD1, survivin, c-Myc,* and B-cell lymphoma 2 (*Bcl-2*), and enhanced *Bax* expression [56].

Ginger also exhibits cytotoxic activity against other types of cancer, such as breast, cervical, liver, and pancreatic cancer. An in vitro study revealed that 6-gingerol could inhibit the growth of HeLa human cervical adenocarcinoma cells, and it induced cell cycle arrest in the G_0_/G_1_-phase by decreasing the protein levels of cyclin A and cyclin D1. Apoptosis in Hela cells was induced by increasing the expression of caspase and inhibiting mammalian target of rapamycin (mTOR) signaling [65]. Besides, ginger extract protected against breast cancer in mice through the activation of 5’adenosine monophosphate-activated protein kinase (AMPK) and the downregulation of cyclin D1. The extract promoted apoptosis via an increase in the expression of the tumor suppressor gene *p53* and a decrease in the level of NF-κB in tumor tissue [58]. Additionally, 10-gingerol was found to be potent in inhibiting human and mouse breast carcinoma cell growth. It reduced cell division and induced S phase cell cycle arrest and apoptosis [66]. Moreover, fluorescent carbon nanodots (C-dots) prepared from ginger effectively controlled tumor growth in nude mice, where the tumor was caused by HepG2 human hepatocellular carcinoma cells. The in vitro experiment found that C-dots increased the content of ROS in the HepG2 cells, which upregulated the expression of *p53* and promoted apoptosis [67]. Furthermore, ginger extract and 6-shogaol suppressed the growth of human pancreatic cancer cells and led to ROS-mediated and caspase-independent cell death. Ginger extract suppressed tumor growth from pancreatic cancer in both a peritoneal dissemination model and an orthotopic model of mice without serious adverse effects [68].

Experimental studies have demonstrated that ginger can prevent and treat several types of cancer, such as colorectal, prostate, breast, cervical, liver, and pancreatic cancer (Table 4). The anticancer mechanisms mainly involve the induction of apoptosis and the inhibition of the proliferation of cancer cells.

### 2.6. Neuroprotection

Some individuals, especially elderly people, have a high risk for neurodegenerative diseases, such as Alzheimer’s disease (AD) and Parkinson’s disease (PD) [69]. Recently, many investigations have revealed that ginger positively affects memory function and exhibits anti-neuroinflammatory activity, which might contribute to the management and prevention of neurodegenerative diseases [70,71].

The results from a lipopolysaccharide (LPS)-activated BV2 microglia culture model revealed that 10-gingerol was responsible for the strong anti-neuroinflammatory capacity of fresh ginger. It inhibited the expression of proinflammatory genes by blocking NF-κB activation, which led to a decline in the levels of NO, IL-1β, IL-6, and TNF-α [7]. Additionally, in mice with scopolamine-induced memory deficits, ginger extract could ameliorate the cognitive function of mice, which was assessed by a novel object recognition test. Further experiments in mouse hippocampi and rat C6 glioma cells revealed that ginger extract promoted the formation of synapses in the brain through the activation of extracellular signal-regulated kinase (ERK) induced by nerve growth factor (NGF) and cyclic AMP response element-binding protein (CREB) [69]. Another study found that 6-shogaol exhibited neuroprotective activity by activating Nrf2, scavenging free radicals, and elevating the levels of several phase II antioxidant molecules, such as NQO1 and HO-1, in neuron-like rat pheochromocytoma PC12 cells [32]. In addition, 6-dehydrogingerdione exhibited cytoprotection against neuronal cell damage induced by oxidative stress. It could effectively scavenge various free radicals in PC12 cells [72]. 

In a mouse model of AD induced by amyloid β_1–42_ plaque, fermented ginger ameliorated memory impairment by protecting neuronal cells in mouse hippocampi, and it increased the levels of presynaptic and postsynaptic proteins [71]. In addition, ginger extract had protective effects against AD in rats, and a high dose of ginger extract decreased latency in showing significant memory deficits, as well as the levels of NF-κB, IL-1β, and MDA [73]. Moreover, 6-shogaol could alleviate cognitive dysfunction in mice with AD by inhibiting inflammatory responses, upregulating the level of NGF, and enhancing synaptogenesis in the brain [74]. Furthermore, in rat mesencephalic cells treated with 1-methyl-4-phenylpyridinium (MPP^+^), 6-shogaol improved the amount of tyrosine hydroxylase-immunoreactive (TH-IR) neurons and inhibited the levels of TNF-α and NO. Treatment with 6-shogaol ameliorated motor coordination and bradykinesia in vivo in PD [70].

The above studies found that ginger and its bioactive compounds, such as 10-gingerol, 6-shogaol, and 6-dehydrogingerdione, exhibited protective effects against AD and PD. The antioxidant and anti-inflammatory activities of ginger contributed to neuroprotection.

### 2.7. Cardiovascular Protection

Cardiovascular diseases have been considered to be a leading cause of premature death, and 17.9 million people die per year [75]. Dyslipidemia and hypertension are known to be risk factors for cardiovascular diseases, including stroke and coronary heart disease [8,76]. A series of studies has shown that ginger can decrease the levels of blood lipids and blood pressure [77,78], contributing to protection from cardiovascular diseases.

Ginger extract reduced the body weight of rats fed a high-fat diet and enhanced the level of serum high-density lipoprotein-cholesterol (HDL-C), a protective factor against coronary heart disease. Besides, ginger extract increased the levels of apolipoprotein A-1 and lecithin-cholesterol acyltransferase mRNA in the liver, which was related to high-density lipoprotein (HDL) formation [79]. Additionally, total cholesterol (TC) and LDL concentrations were decreased by ginger extract in rats fed a high-fat diet, and the level of HDL increased through the combined application of aerobic exercise and ginger extract [76]. Moreover, ginger extract could reduce the levels of plasma TC, triglyceride (TG), and very low-density lipoprotein (VLDL) cholesterol in high-fat diet rats. The mechanism was related to higher liver expression of peroxisome proliferator-activated receptors (PPARα and PPARγ), which were related to atherosclerosis [78]. 

Vascular smooth muscle cell proliferation is a process in the pathogenesis of cardiovascular diseases. In an in vitro study, 6-shogaol exerted antiproliferative effects through increasing the number of cells in the G_0_/G_1_ phase and activating the Nrf2 and HO-1 pathways [80]. In addition, ginger decreased the activities of angiotensin-1 converting enzyme (ACE) and arginase and increased the level of NO, a well-known vasodilator molecule. Thus, blood pressure decreased in hypertensive rats pretreated with ginger [8]. Besides, ginger protected against hypertension-derived complications by decreasing platelet adenosine deaminase (ADA) activity and increasing the level of adenosine, which prevented platelet aggregation and promoted vasodilation in hypertensive rats [77]. Moreover, ginger extract exhibited vasoprotective effects on porcine coronary arteries by suppressing NO synthase and cyclooxygenase [81]. Furthermore, a cross-sectional study found that the probability of hypertension and coronary heart disease declined when a daily intake of ginger was increased [82].

Generally, ginger has exhibited cardiovascular protective effects by attenuating hypertension and ameliorating dyslipidemia, such as in the improvement of HDL-C, TC, LDL, TG, and VLDL.

### 2.8. Antiobesity Activity

Obesity is a risk factor for many chronic diseases, such as diabetes, hypertension, and cardiovascular diseases [83]. Several studies have reported that ginger is effective in the management and prevention of obesity [9,84]. 

In 3T3-L1 preadipocyte cells, gingerenone A exhibited a greater inhibitory effect on adipogenesis and lipid accumulation than gingerols and 6-shogaol. Gingerenone A could also modulate fatty acid metabolism via the activation of AMPK in vivo, attenuating diet-induced obesity [9]. In cultured skeletal muscle myotubes, 6-shogaol and 6-gingerol could increase peroxisome proliferator-activated receptor δ (PPARδ)-dependent gene expression, and this resulted in the enhancement of cellular fatty acid catabolism [83]. In addition, both ginger and orlistat reduced the body weight and lipid profile of high-fat diet rats, while ginger had a greater effect on increasing the level of HDL-C than orlistat did [84]. In a randomized, double-blind, and placebo-controlled study, obese women receiving 2 g of ginger powder daily had a decreased body mass index (BMI) [85]. Moreover, the intake of dried ginger powder could reduce respiratory exchange ratios and promote fat utilization by increasing fat oxidation in humans [86].

Ginger and its bioactive constituents, including gingerenone A, 6-shogaol, and 6-gingerol, have shown antiobesity activity, with the mechanisms mainly related to the inhibition of adipogenesis and the enhancement of fatty acid catabolism.

### 2.9. Antidiabetic Activity

Diabetes mellitus is known as a severe metabolic disorder caused by insulin deficiency and/or insulin resistance, resulting in an abnormal increase in blood glucose. Prolonged hyperglycemia could accelerate protein glycation and the formation of advanced glycation end products (AGEs) [87]. Many research works have evaluated the antidiabetic effect of ginger and its major active constituents [88].

An in vitro experiment resulted in both 6-shogaol and 6-gingerol preventing the progression of diabetic complications, and they inhibited the production of AGEs by trapping methylglyoxal (MGO), the precursor of AGEs [87]. Additionally, 6-gingerol reduced the levels of plasma glucose and insulin in mice with high-fat diet-induced obesity. Nε-carboxymethyl-lysine (CML), a marker of AGEs, was decreased by 6-gingerol through Nrf2 activation [88]. In 3T3-L1 adipocytes and C2C12 myotubes, 6-paradol and 6-shogaol promoted glucose utilization by increasing AMPK phosphorylation. In addition, in a mouse model fed a high-fat diet, 6-paradol significantly reduced the level of blood glucose [10]. In another study, 6-gingerol facilitated glucose-stimulated insulin secretion and ameliorated glucose tolerance in type 2 diabetic mice by increasing glucagon-like peptide 1 (GLP-1). Besides, 6-gingerol treatment activated glycogen synthase 1 and increased cell membrane presentation of glucose transporter type 4 (GLUT4), which increased glycogen storage in skeletal muscles [89]. Furthermore, the consumption of ginger could reduce the levels of fasting plasma glucose, glycated hemoglobin A (HbA1_C_), insulin, TG, and TC in patients with type 2 diabetes mellitus (DM2) [90]. Moreover, ginger extract treatment improved insulin sensitivity in rats with metabolic syndrome, which might have been relevant to the energy metabolism improvement induced by 6-gingerol [91]. In addition, ginger extract alleviated retinal microvascular changes in rats that had diabetes induced by streptozotocin. Ginger extract could reduce the levels of NF-κB, TNF-α, and vascular endothelial growth factor in the retinal tissue [92]. In a randomized, double-blind, and placebo-controlled trial, the ingestion of ginger decreased the levels of insulin, low-density lipoprotein cholesterol (LDL-C), and TG; decreased the homeostasis model assessment index; and increased the quantitative insulin sensitivity check index in patients with DM2 [93].

The studies have demonstrated that ginger and its bioactive compounds could protect against diabetes mellitus and its complications, probably by decreasing the level of insulin, but increasing the sensitivity of insulin.

### 2.10. Antinausea and Antiemetic Activities

Ginger has been traditionally used to treat gastrointestinal symptoms, and recent research has demonstrated that ginger could effectively alleviate nausea and emesis [11,94,95].

In a clinical trial, inhaling ginger essence could attenuate nausea intensity and decrease emesis episodes two and six hours after a nephrectomy in patients [96]. In addition, dried ginger powder treatment reduced episodes of intraoperative nausea in elective cesarean section patients [97]. Moreover, nausea and emesis are common side effects of chemotherapy [98]. The activation of vagal afferent mediated by serotonin (5-HT) is crucial in the mechanism of emesis. An in vitro experiment revealed that 6-shogaol, 6-gingerol, and zingerone inhibited emetic signal transmission in vagal afferent neurons by suppressing the 5-HT receptor, and 6-shogaol had the strongest inhibitory efficacy [99]. Furthermore, ginger extract alleviated chemotherapy-induced nausea and emesis by suppressing the activation of 5-HT receptors in enteric neurons [11]. In a double-blind, randomized, and placebo-controlled trial, supplementation with ginger could improve the nausea-related quality of life in patients after chemotherapy [94]. Moreover, ginger alleviated the nausea induced by antituberculosis drugs and antiretroviral therapy, and it reduced the frequency of mild, moderate, and severe episodes of nausea in patients [100,101].

Previous results have shown that ginger could attenuate pregnancy-induced nausea and emesis and motion sickness, while recent studies have focused on the preventive efficacy of ginger on postoperative and chemotherapy-induced nausea and emesis [102].

### 2.11. Protective Effects against Respiratory Disorders

Natural herbal medicines have a long history of application in the treatment of respiratory disorders such as asthma, and ginger is one of these remedies [12,103]. Ginger and its bioactive compounds have exhibited bronchodilating activity and antihyperactivity in several studies [104].

Ginger induced significant and rapid relaxation in the isolated human airway smooth muscle. In results from guinea pig and human tracheas models, 6-gingerol, 8-gingerol, and 6-shogaol could lead to the rapid relaxation of precontracted airway smooth muscle. The nebulization of 8-gingerol attenuated airway resistance via a reduction in Ca^2+^ influx in mice [12]. In another study, 6-gingerol, 8-gingerol, and 6-shogaol promoted β-agonist-induced relaxation in human airway smooth muscle via the suppression of phosphodiesterase 4D [103]. In addition, ginger ameliorated allergic asthma by reducing allergic airway inflammation and suppressed Th2-mediated immune responses in mice with ovalbumin-induced allergic asthma [105]. Moreover, the water-extracted polysaccharides of ginger could decrease times of coughing, which was induced through citric acid in guinea pigs [106]. Besides, ginger oil and its bioactive compounds, including citral and eucalyptol, inhibited rat tracheal contraction induced by carbachol in rats [104]. Furthermore, in patients with acute respiratory distress syndrome (ARDS), an enteral diet with rich ginger contributed to gas exchange and reduced the duration of mechanical ventilation [107].

The above results indicate that ginger and its bioactive constituents, including 6-gingerol, 8-gingerol, 6-shogaol, citral, and eucalyptol, have protective effects against respiratory disorders, at least mediating them through the induction of relaxation in airway smooth muscle and the attenuation of airway resistance and inflammation.

### 2.12. Other Bioactivities of Ginger

**Figure 3:**
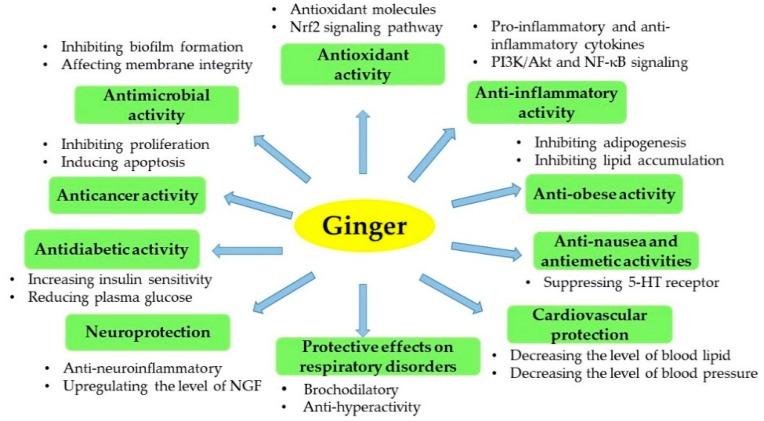
An overview of the bioactivities of ginger.

Apart from the bioactivities mentioned above (Figure 3), ginger has other beneficial effects, such as hepatoprotective and antiallergic effects [108,109].

In a rat nephropathy model induced by gentamicin, gingerol dose-dependently ameliorated renal function and reduced lipid peroxidation and nitrosative stress. Gingerol also increased the levels of GSH and the activity of superoxide dismutase (SOD) [110]. Additionally, ginger extract ameliorated histological and biochemical alterations in the radiation-induced kidney damage of rats through antioxidant and anti-inflammatory activities [111]. Furthermore, liver histological results showed that ginger essential oil reduced lipid accumulations in the liver of obese mice fed a high-fat diet. Ginger essential oil could protect against steatohepatitis by enhancing antioxidant capacity and reducing inflammatory responses in the liver [109]. In another study with mice fed an alcohol-containing liquid diet, ginger essential oil ameliorated alcoholic fatty liver disease by decreasing the levels of AST, ALT, TG, and TC and increasing liver antioxidant enzyme activity, such as catalase and SOD [112]. To our knowledge, there has been no literature reporting the liver toxicity of ginger up to now. Additionally, in a mouse model of allergic rhinitis induced by ovalbumin (OVA), a ginger diet attenuated the severity of sneezing and nasal rubbing and inhibited the infiltration of mast cells into nasal mucosa as well as the secretion of serum immunoglobulin E. The in vitro study indicated that 6-gingerol could alleviate allergic rhinitis by reducing cytokine production for T cell activation and inhibiting the activation of B cells and mast cells [108]. Moreover, treatment with ginger could reduce blood loss in women with heavy menstrual bleeding [113]. In a double-blinded randomized clinical trial, treatment with ginger powder alleviated a common migraine attack and had fewer clinical adverse effects than the clinical medicine sumatriptan [114].

It is interesting to note that several plants in Zingiberaceae have also attracted increasing attention, such as *Curcuma longa* L. (turmeric), *Zingiber officinale* Roscoe (ginger), and *Alpinia zerumbet* (shell ginger) [115]. In a previous paper, we reviewed the bioactivities of curcumin (main active component of *Curcuma longa*) [116], and a comparison between ginger and shell ginger is given in Table 5. Shell ginger has exhibited similar biological activities to ginger, including antioxidant, anti-inflammatory, antimicrobial, anticancer, cardiovascular protective, antiobesity, and antidiabetic activities [115]. Differently, ginger has also been reported to have neuroprotective, respiratory protective, antinausea, and antiemetic activities, while shell ginger might contribute to longevity. In particular, shell ginger has been found to play an important contributory role in the longevity of people in Okinawa [115]. 

## 3. Conclusions

In conclusion, ginger contains diverse bioactive compounds, such as gingerols, shogaols, and paradols, and possesses multiple bioactivities, such as antioxidant, anti-inflammatory, and antimicrobial properties. Additionally, ginger has the potential to be the ingredient for functional foods or nutriceuticals, and ginger could be available for the management and prevention of several diseases such as cancer, cardiovascular diseases, diabetes mellitus, obesity, neurodegenerative diseases, nausea, emesis, and respiratory disorders. In the future, more bioactive compounds in ginger could be isolated and clearly identified, and their biological activities and related mechanisms of action should be further investigated. Notably, well-designed clinical trials of ginger and its various bioactive compounds are warranted to prove its efficacy against these diseases in human beings.

## Figures and Tables

**Table 1:** The antioxidant activity and potential mechanisms of ginger.

Constituent	Study Type	Subjects	Dose	Potential Mechanisms	Ref.
6-shogaol	In vivo	HCT-116 human colon cancer cells	20 μM	Increasing the intracellular GSH/GSSG ratio; decreasing the level of ROS; upregulating the expression of *AKR1B10, FTL, GGTLA4, HO-1, MT1, GCLC,* and *GCLM* genes	[33]
	In vitro	Wild-type and Nrf2^−/−^ C57BL/6J mice	100 mg/kg	Upregulating the expression of *MT1, HO-1*, and *GCLC*	
Ginger oleoresin	In vitro	Human mesenchymal stem cells	100 μg/mL	Reducing ROS production; inducing the translocation of Nrf2 to the cell nucleus; activating *HO-1* and *NQO1* gene expression	[14]
Ginger phenylpropanoids	In vitro	BJ foreskin fibroblasts	40 μg/mL	Increasing Nrf2 activity and the level of GSTP1	[15]
6-gingerol-rich fraction	In vivo	Female Wistar rats	50 and 100 mg/kg	Reducing the levels of H_2_O_2_ and MDA; increasing the activities of antioxidant enzymes and the level of GSH	[25]
Ginger extract	In vivo	Male Wistar albino rats	100 mg/kg	Reducing the level of MDA; preventing the depletion of catalase activity and GSH content	[34]
	In vitro	C28I2 human chondrocyte cells	5 and 25 μg/mL	Increasing the gene expression of antioxidant enzymes; reducing the content of ROS and lipid peroxidation	[30]
	In vitro	HT1080 human fibrosarcoma cells	200 and 400 μg/mL	Reducing the generation of ROS	[31]
	In vitro	Rat heart homogenates	78–313 μg/mL	Decreasing the level of MDA	[29]

GSSG, glutathione disulfide; MT1, metallothionein 1; GSTP1, glutathione S-transferase P1; MDA, malondialdehyde; Ref, reference.

**Table 2:** Anti-inflammatory activity and potential mechanisms of ginger.

Constituent	Study Type	Subjects	Dose	Potential Mechanisms	Ref.
6-shogaol	In vitro	HT-29/B6 and Caco-2 human intestinal epithelial cells	100 μM	Inhibiting the PI3K/Akt and NF-κB signaling pathways	[37]
6-shogaol and 6-gingerol, 6-dehydroshogaol	In vitro	RAW 264.7 mouse macrophage cells	2.5, 5, and 10 μM	Inhibiting the production of NO and PGE_2_	[36]
6-gingerol-rich fraction	In vivo	Female Wistar rats	50 and 100 mg/kg	Increasing the levels of myeloperoxidase, NO, and TNF-α	[25]
GDNPs 2	In vivo	Female C57BL/6 FVB/NJ mice	0.3 mg	Increasing the levels of IL-10 and IL-22; decreasing the levels of TNF-α, IL-6, and IL-1β	[4]
Ginger extract and zingerone	In vivo	Female BALB/c mice	0.1, 1, 10, and 100 mg/kg	Inhibiting NF-κB activation and decreasing the level of IL-1β	[38]
Ginger extract	In vivo	C57BL6/J mice	50 mg/mL	Inhibiting the production of TNF-α; Activating Akt and NF-κB	[39]

NO, nitric oxide; PGE_2_, prostaglandin E_2_; TNF-α, tumor necrosis factor α; GDNPs 2, nanoparticles derived from edible ginger.

**Table 3:** Antimicrobial activity and potential mechanisms of ginger.

Constituent	Study Type	Subjects	Dose	Potential Mechanisms	Ref.
Ginger essential oil	In vitro	*Fusarium verticillioides*	500, 1000, 2000, 3000, 4000, and 5000 μg/mL	Reducing ergosterol biosynthesis; affecting membrane integrity; decreasing the production of fumonisin B1 and fumonisin B2	[51]
	In vitro	*Aspergillus flavus*	5, 10, 15, 20, 25, 50, 100, and 150 μg/mL	Reducing ergosterol biosynthesis; affecting membrane integrity; inhibiting the production of aflatoxin	[50]
Gingerenone-A and shogaol	In vitro	*Staphylococcus aureus*	25, 50, and 75 μg/mL	Inhibiting the activity of 6-hydroxymethyl-7, 8-dihydropterin pyrophosphokinase	[49]
Ginger extract	In vitro	*Pseudomonas aeruginosa*	50, 100, 150, and 200 μg/mL	Affecting membrane integrity; inhibiting biofilm formation	[46]
	In vitro	*Streptococcus mutans*	8, 16, 32, 64, and 128 μg/mL	Inhibiting biofilm formation, glucan synthesis, and adherence	[48]
	In vitro	HEp-2 human larynx epidermoid carcinoma cells and A549 human lung carcinoma cells with HRSV	10, 30, 100, and 300 μg/mL	Blocking viral attachment and internalization	[52]

HRSV, human respiratory syncytial virus.

**Table 4:** Cytotoxic activity and potential mechanisms of ginger.

Constituent	Study Type	Subjects	Dose	Potential Mechanisms	Ref.
6-shogaol	In vitro	LNCaP, DU145, and PC-3 human prostate cancer cells	10, 20, and 40 μM	Inducing apoptosis; inhibiting STAT3 and NF-κB signaling; downregulating the expression of *cyclin D1, survivin, c-Myc,* and *Bcl2*	[57]
6-gingerol	In vitro	HeLa human cervical adenocarcinoma cells	60, 100, and 140 μM	Inducing cell cycle arrest in the G_0_/G_1_-phase; decreasing the levels of cyclin A, cyclin D1, and cyclin E1; increasing the expression of caspase; inhibiting the mTOR signaling pathway	[65]
10-gingerol	In vitro	Human and mouse breast carcinoma cells	50, 100, and 200 μM	Inhibiting cell growth; reducing cell division; inducing S phase cell cycle arrest and apoptosis	[66]
6-gingerol, 10-gingerol, 6-shogaol, and 10-shogaol	In vitro	PC-3 human prostate cancer cells	1,10, and 100 μM	Inhibiting prostate cancer cell proliferation; downregulating the expression of MRP1and GSTπ	[59]
GDNPs 2	In vivo	Female C57BL/6 mice	0.3 mg	Suppressing the expression of cyclin D1; inhibiting intestinal epithelial cell proliferation	[4]
Ginger extract	In vitro	HT29 human colorectal adenocarcinoma cells	2–10 mg/mL	Promoting apoptosis; upregulating the caspase 9 gene; downregulating *KRAS, ERK, Akt*, and *Bcl-xL*	[60]
	In vivo	Female Swiss albino mice	100 mg/kg	Activating AMPK; decreasing the expression of cyclin D1 and the level of NF-κB; increasing the expression of *p53*	[58]
Ginger extract with alginate beads	In vivo	Male Wistar rats	50 mg/kg	Increasing the activity of NADH dehydrogenase and succinate dehydrogenase	[61]
Ginger extract-based fluorescent carbon nanodots	In vitro	HepG2 human hepatocellular carcinoma cells	1.11 mg/mL	Increasing the level of ROS; upregulating the expression of *p53*; promoting apoptosis	[67]

STAT3, signal transducer and activator of transcription 3; Bcl-2, B-cell lymphoma 2; mTOR, mammalian target of rapamycin; MRP1, multidrug resistance associated protein 1; GSTπ, glutathione-S-transferase; AMPK, 5’adenosine monophosphate-activated protein kinase; NF-κB, nuclear factor kappa light chain-enhancer of activated B cells.

**Table 5:** The comparison between ginger and shell ginger.

Items	Ginger	Shell Ginger	Ref.
Scientific name	*Zingiber officinale* Roscoe	*Alpinia zerumbet* (Pers.) B.L. Burtt & R.M. Sm.	[115,117]
Family and genus	Zingiberaceae family and *Zingiber* genus	Zingiberaceae family and *Alpinia* genus	[115,117]
Edible parts	Rhizomes	Leaves and rhizomes	[8,115]
Bioactive compounds	Gingerols, shogaols, paradols, and essential oils	Dihydro-5,6-dehydrokawain, 5,6-dehydrokawain, essential oils, and flavonoids	[2,44,115]
Biological activities	Antioxidant, anti-inflammatory, antimicrobial, anticancer, cardiovascular protective, antiobesity, antidiabetic, neuroprotective, respiratory protective, antinausea, and antiemetic activities	Antioxidant, anti-inflammatory, antimicrobial, anticancer, cardiovascular protective, antiobesity, antidiabetic activities, longevity	[3,4,5,6,7,8,9,10,11,12,115]
